# OCT4 and MENA immunoprofiling in salivary mucoepidermoid carcinoma

**DOI:** 10.1186/s13000-025-01665-8

**Published:** 2025-05-27

**Authors:** Omnia Samir, Doaa A. Farag, Khadiga M. Ali, Lawahez El. M. Ismail

**Affiliations:** 1 Department of Oral Pathology, Faculty of Dentistry, Mansoura University, Mansoura, Egypt; 2 Department of Pathology, Faculty of Medicine, Mansoura University, Mansoura, Egypt

**Keywords:** Mucoepidermoid carcinoma, OCT4, MENA, Wnt/β-catenin signaling pathway, Immunohistochemistry

## Abstract

**Background:**

Mucoepidermoid carcinoma (MEC) emblematizes the predominant malignant salivary gland neoplasm, characterized by its heterogeneous morphological features and diverse clinical representations. The expression patterns and prognostic significance of Octamer transcription factor 4 (OCT4) and Mammalian-enabled (MENA) protein in MEC perdure are incompletely described.

**Methods:**

Immunohistochemical analysis was performed on 46 archival MEC specimens and 5 normal salivary-gland controls. OCT4 and MENA staining were assessed histomorphometrically and correlated with clinicopathological parameters. Statistical analysis comprised Monte Carlo and Spearman’s correlation tests.

**Results:**

OCT4 revealed selective cytoplasmic immunoreactivity in intermediate and epidermoid cells, without nuclear positivity. Strong OCT4 expression predominated in low-grade (66.7%), while high-grade MEC exhibited variable immunoreactivity, with 53% showing weak expression. No significant correlation was found between OCT4 expression and clinical or pathological data. MENA showed cytoplasmic and membranous immunolocalization, with expression patterns correlated significantly with age (*p* = 0.015), tumor size (*p* = 0.012), clinical stage (*p* = 0.004), and histological grading (*p* = 0.001). Spearman’s correlation analysis revealed a weak, non-significant association between OCT4 and MENA expression (*r* = 0.05, *p* = 0.744).

**Conclusions:**

The differential expression patterns of OCT4 and MENA in MEC prognosticate distinct regulatory mechanisms. While OCT4 cytoplasmic expression may presage early involvement in carcinogenesis, MENA cellular expression portends potentially independent molecular pathways, possibly encompassing subnetworks in the Wnt/β-catenin and TGF-β signaling cascades. MENA may serve as a biomarker for predicting the aggressive behavior of MEC.

**Supplementary Information:**

The online version contains supplementary material available at 10.1186/s13000-025-01665-8.

## Introduction

Mucoepidermoid carcinoma (MEC) is the most common malignant salivary gland tumor (SGT) in adults and children, accounting for 10–15% of all SGT and 30% of all salivary malignancies. Due to its rich morphologies and variable prognostic indicators, it remains diagnostically challenging. MEC instantiates a tripartite cellular composition: mucous-secreting, squamous (epidermoid), and intermediate cells [1]. The neoplasm subsumes a three-tiered grading schema, wherein modified Healey, Armed Forces Institute of Pathology (AFIP), Brandwein, and Katabi scoring systems (MSKCC) partly conflict [2]. The clinical staging and histological grade perdure as cardinal prognostic harbingers. Yet, the etiopathogenesis of MEC is unclear. Recently, cancer stem cells (CSCs) with self-renewal and multilineage differentiation properties may contribute to tumor development and progression [3, 4].

Octamer transcription factor 4 (OCT4), encoded by the *POU5F1* gene and appertaining to the POU-domain (Pit-Oct-Unc) transcription factor family, superintends embryonic stem cell and CSC pluripotency maintenance [5]. Further, OCT4 impinges upon neoplastic progression via cellular proliferation and epithelial-mesenchymal transition (EMT), colligating with Wnt/β-catenin and Notch signaling cascades [5, 6]. While OCT4 overexpression often portends poor outcomes [7–10], its prognostic import remains contentious [11–13], with OCT4 overexpression corresponding to better prognosis in hypopharyngeal squamous cell carcinoma [14]. This controversy is potentially attributed to its discrete isoforms (OCT4A, OCT4B, and OCT4B1), which differentially modulate stemness and differentiation [15].

Mammalian-enabled (MENA) protein is an ENA/ VASP family member, encoded by the ENAH gene on chromosome 1. MENA is a cytoskeletal regulatory protein that is essential in forming invasive membrane protrusions and regulates actin dynamics in cell motility and adhesion [16]. It also interacts with various signaling pathways, including Wnt/β-catenin and TGF-β, which influence tumor progression and cell motility, highlighting its significance in cancer development and metastasis [17–19]. MENA overexpression presages increased invasiveness and diminished survival across multiple malignancies, such as thyroid, gastric, and oral squamous cell carcinoma [19–21]. Therefore, OCT4 and MENA expression in MECs is investigated in relation to their clinical behavior.

## Materials and methods

### Case selection

Forty-six archived MEC specimens preserved in formalin-fixed paraffin blocks (FFEB), contrasted against five non-neoplastic salivary parenchyma specimens excised during mucocele resection. Clinicopathological parameters were extrapolated from medical documentation and stratified per AJCC [22]. The research was approved by the Institutional Ethical Committee (code: M0108023OP).

### Immunohistochemistry

Quadrimicrometer sections underwent xylene deparaffinization, descending ethanol gradients, and aqueous equilibration. Endogenous peroxidase quenching utilized 0.5% H_2_O_2_ in methanolic solution using the Avidin-Biotin complex method. Primary antibodies (OCT4 1:75 dilution, TÜV Rheinland Group; MENA 1:75 dilution, Elabscience^®^) were applied and chromogenized using 3.3’ diaminobenzidine-4HCL. Then, overnight post-PBS lavage was implemented. OCT4 manifestation presented as nucleocytoplasmic brown staining, while MENA exhibited cytomembranous localization. Positive and negative controls were run in parallel.

### Histomorphometry

Initial microscopic examination at 40x magnification identified immunoreactive regions. Subsequent digital microdocumentation at 400x magnification encompassed four discrete fields. Image J software quantified immunoreactivity through optical density stratification: negligible (< 0.2), weak (0.3–0.7), moderate (0.7–1.1), and strong (> 1.1). For OCT4, the percentage of positive tumor cells was scored as follows: 1 (< 10%), 2 (10-50%), and 3 (> 50%) [23, 24]. The final IHC scores for OCT4, obtained by multiplying the proportion of positive cells by intensity, ranged from 0 to 9. Scores 0, 1, and 2 were considered not or weakly positive, 3 and 4 moderately positive, and 6 and 9 strongly positive [24]. For MENA, the percentage of positive tumor cells was scored as: 0 (no positive cells), 1 (1-25%), 2 (26-50%), 3 (50-75%), and 4 (> 75%). The final IHC scores for MENA also obtained by multiplication, ranged from 0 to 12, with expression levels defined as: negative (0), weakly positive (1–4), moderately positive (5–8), and strongly positive (9–12) [20, 21].

### Statistical analysis

SPSS software enabled using Monte Carlo tests for negotiating parametric data, while Spearman’s rank correlation coefficient assessed non-parametric continuous and ordinal co-variates.

## Results

As shown in Table 1. This study examined 46 MECs graded using the modified Haley system (not AFIB, Brandwein or MSKCC) into 15 low-grade, 14 intermediate-grade, and 17 high-grade. Patient ages ranged from 14 to 78 years (mean 47.47 ± 18.31 years). Female patients predominated (33 cases, 71.7%), creating a female-to-male ratio of 2.5:1.

**Table 1:** The distribution of histopathological grades of MEC among the clinical data of the MEC cases studied:

		MODIFIED HALEY		
	Total *N* = 46 (%)	Low grade *N* = 15 (%)	Intermediate grade *N* = 14 (%)	High grade *N* = 17 (%)
**Age /years** **Mean / (SD)**	47.47 (± 18.31).			
**Range**				
≤ 45 45–60 > 60	22 (47.8) 11 (24) 13 (28.2)	9(60) 5(33.3) 1(6.7)	6(42.9) 5(35.7) 3(21.4)	7(41.2) 1(5.9) 9(52.9)
**Sex**				
Female Male	33 (71.7) 13 (28.3)	12(80) 3(20)	11(78.6) 3(21.4)	10(58.8) 7(41.2)
**Site**				
parotid Submandibular Minor glands Intraosseous	31 (67.4) 2 (4.3) 11 (24) 2 (4.3)	10(66.7) 1(6.6) 4 (26.7) 0	10(71.4) 1(7.1) 3 (21.5) 0	11(64.7) 0 4 (23.5) 2 (11.8)
**Stage**				
I II III IV	17 (37.0) 8 (17.4) 16 (34.8) 4 (10.9)	7(46.7) 5(33.3) 3(20) 0	7(50.0) 2(14.3) 4(28.6) 1(7.1)	3(17.6) 1(5.9) 9(52.9) 4(23.5)
**Lymph node**				
N0 N1 N2	40 (87.0) 2 (4.3) 4 (8.7)	15(100) 0 0	12(85.7) 1(7.1) 1(7.1)	13(76.5) 1(5.9) 3(17.6)
**Tumor size**				
T1 T2 T3 T4	17 (37.0) 9 (19.6) 16 (34.8) 4 (8.6)	7(46.7) 5(33.3) 3(20) 0	7(50) 2(14.3) 5(35.7) 0	3(17.60 2(11.8) 8(47.1) 4(23.5)

Clinically, the parotid gland was the most common tumor site (31 cases, 67.4%), followed by minor salivary glands (11 cases, 24%), with half of the minor gland cases occurring in the palate. Two cases (4.3%) were found in the submandibular gland, and two (4.3%) were intraosseous. Most tumors presented as either T1 (17 cases, 37%) or T3 (16 cases, 34.8%), with high-grade MEC more frequently associated with T3 staging and low-grade variants predominantly found in T1. The majority of cases (40 cases, 87%) showed no nodal metastasis (N0), while six cases (13%) exhibited nodal lymphatic spread.

In non-neoplastic salivary tissue, OCT4 showed no expression in mucous acinar elements but demonstrated cytoplasmic positivity in ductal epithelia. MENA exhibited immunopositivity in ductal and serous acinar elements, while mucous cells were negative. The controls are shown in Supplemental material.

**Fig. 1:**
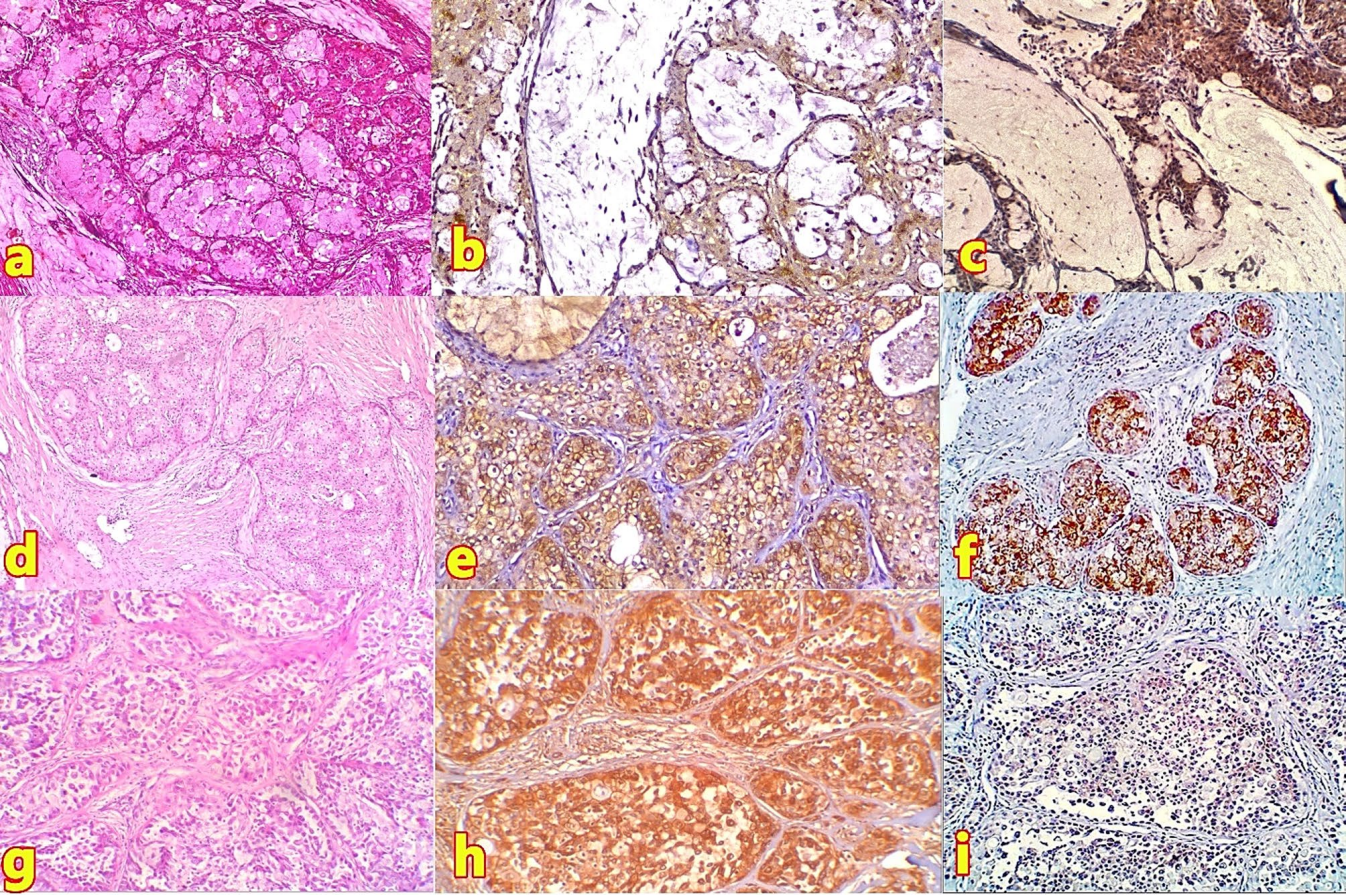
Histopathological and immunohistochemical characteristics of different MEC grades. (**a**) LG-MEC showing predominant cystic architecture lined by mucin-secreting, intermediate, and epidermoid cells (H&E, 40×). (**b**) Same case demonstrating weak cellular MENA expression in epidermoid and intermediate cells, mucinous cells were not reactive (IHC, 100×). (**c**) Same case displaying diffuse cytoplasmic OCT4 expression in epidermoid and intermediate components, mucinous cells were not reactive (IHC, 100×). (**d**) Intermediate-grade MEC featuring minimal cystic spaces with mild cellular pleomorphism (H&E, 40×). (**e**) Same case showing moderate cellular MENA expression (IHC, 100×). (**f**) Same case exhibiting moderate cytoplasmic OCT4 expression (IHC, 100×). (**g**) High-grade MEC presenting predominantly solid nests of epidermoid cells and small amphophilic-to-eosinophilic ductal cells with oval nuclei that show increased pleomorphism (H&E, 100×). (**h**) Same case demonstrating strong block-positive MENA (IHC, 100×). (**i**) Same case showing weak OCT4 expression patterns (IHC, 100×)

Morphologically, LG-MEC (Fig. 1a) displayed cystic spaces lined by mucin-secreting, intermediate, and epidermoid cells, with occasional solid clusters. Intermediate-grade tumors (Fig. 1d) showed decreased cystic spaces with mild to moderate cellular pleomorphism and infrequent mitotic figures. High-grade specimens (Fig. 1g) presented predominantly solid epidermoid cell arrangements with increased pleomorphism and mitotic activity.

LG-MEC showed diffuse cytoplasmic OCT4 expression (Fig. 1c) - but no nuclear immunostaining - and predominantly weak MENA immunoreactivity (Fig. 1b) in 86.7% of cases. Intermediate-grade specimens displayed diffuse cytoplasmic OCT4 expression (Fig. 1f), although it is a nuclear transcription factor, and moderate MENA immunoreactivity (Fig. 1e) in 57.2% of cases. High-grade variants exhibited either weak (Fig. 1i) or negative OCT4 immunoreactivity (not shown), denoting that OCT4 is not a marker of stemness/aggressiveness, but strong MENA expression (Fig. 1h) in 94% of cases.

**Fig. 2:**
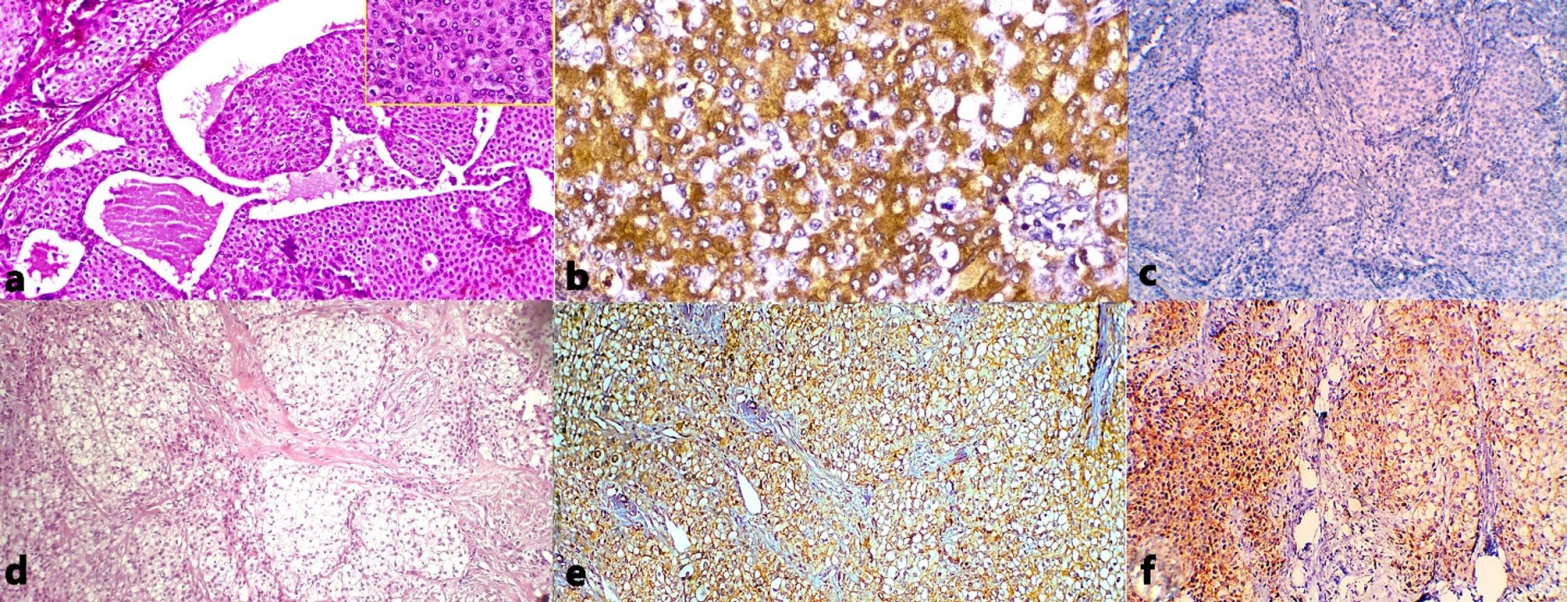
Histopathological and immunohistochemical features of MEC variants. (**a**) Oncocytic variant of intermediate-grade MEC with abundant granular eosinophilic cytoplasm in oncocytes; inset showing mucocytes, characteristic of MEC (H&E, 100×). (**b**) Same case displaying moderate cellular MENA expression (IHC, 200×). (**c**) Same case showing absence of OCT4 expression (IHC, 100×). (**d**) Clear cell variant of high-grade MEC exhibiting nests of clear cells with distinct cell borders (H&E, 100×). (**e**) Same case demonstrating strong cytomembranous MENA immunoreactivity in both clear and epidermoid components (IHC, 100×). (**f**) Same case showing strong cytoplasmic OCT4 positivity in both clear and epidermoid cells (IHC, 100×)

The oncocytic variant of intermediate-grade MEC (Fig. 2a) demonstrated distinctive mucinous features highlighted by Alcian blue staining. This variant showed moderate MENA immunoreactivity (Fig. 2b) but no OCT4 expression (Fig. 2c). The clear cell variant of high-grade MEC (Fig. 2d) exhibited nested cell arrangements and demonstrated strong MENA immunoreactivity (Fig. 2e) as well as strong OCT4 positivity in both epidermoid and clear cells (Fig. 2f). It is unusual for high-grade MEC to be entirely differentiated into a clear-cell-rich phenotype.

Statistical analysis revealed no significant correlation between OCT4 expression and clinical or pathological characteristics. However, MENA expression showed statistically significant associations with age (*p* = 0.015), tumor size (*p* = 0.012), clinical staging (*p* = 0.004), and histopathological grading (*p* = 0.001) ass shown in Table 2. No information about treatment approaches or patient outcomes was accessible.

**Table 2:** Clinicopathological characteristics and marker expression in MEC

Parameter	*n*	OCT4 Expression			MENA Expression		
		Weak/Negative (*n* = 15)	Moderate (*n* = 5)	Strong (*n* = 26)	Weak (*n* = 16)	Moderate (*n* = 11)	Strong (*n* = 19)
Age (years)							
≤ 45	22	5	1	16	8	7	7
45–60	11	4	2	5	7	2	2
> 60	13	6	2	5	1	2	10
p-value		*p* = 0.283			*p* = 0.015*		
Sex							
Female	33	10	2	21	12	9	12
Male	13	5	3	5	4	2	7
p-value		*p* = 0.156			*p* = 0.516		
Site							
Parotid	31	7	4	20	9	9	13
Minor gland	11	5	1	5	6	2	3
Intraosseous	2	2	0	0	0	0	2
Submandibular	2	1	0	1	1	0	1
p-value		*p* = 0.667			*p* = 0.588		
Tumor Size							
T1	17	4	2	11	10	4	3
T2	9	3	2	4	3	4	2
T3	16	4	1	11	3	3	10
T4	4	4	0	0	0	0	4
p-value		*p* = 0.081			*p* = 0.012*		
Lymph Node Status							
N0	40	12	5	23	16	10	14
N1	2	0	0	2	0	1	1
N2	4	3	0	1	0	0	4
p-value		*p* = 0.280			*p* = 0.103		
Stage							
I	17	4	2	11	10	4	3
II	8	3	2	3	3	4	1
III	16	4	1	11	3	3	10
IV	5	4	0	1	0	0	5
p-value		*p* = 0.181			*p* = 0.004*		
Grade							
Low	15	2	3	10	13	2	0
Intermediate	14	4	1	9	3	8	3
High	17	9	1	7	0	1	16
p-value		*p* = 0.148			*p* = 0.001*		

*Statistically significant (*p* < 0.05)

**Fig. 3:**
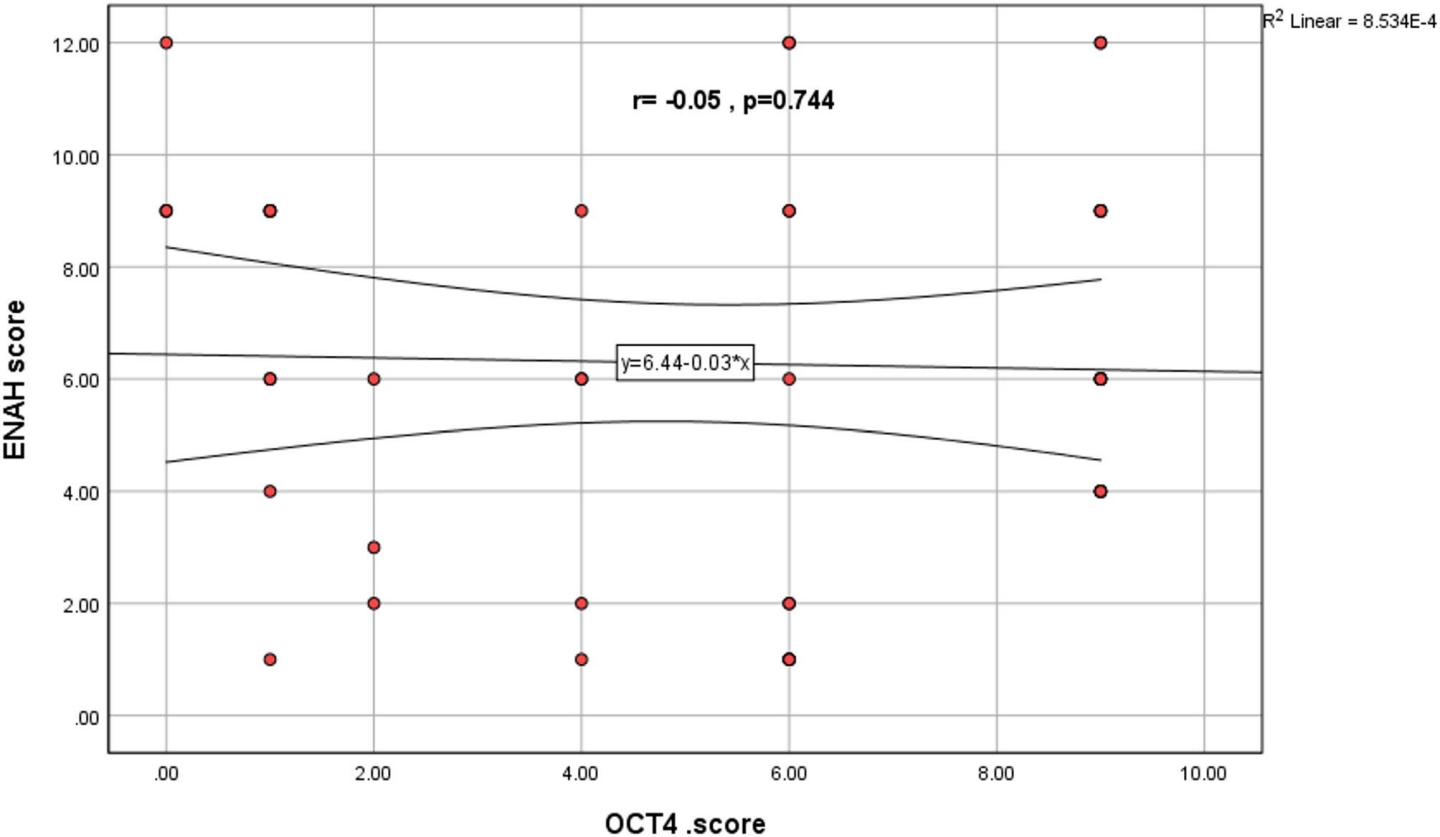
Scatter plot Correlation between different studied two markers

Spearman’s correlation test revealed a weak, statistically non-significant correlation between OCT4 and MENA immunohistochemical expression in salivary gland MEC (*r* = 0.05, *p* = 0.744), indicating that these markers operate independently in MEC pathogenesis as shown in Fig. 3.

## Discussion

Histological grading is essential for predicting tumor behaviour, prognosis, and patient management. While various grading systems exist, no universally accepted one has been established due to subjectivity and tumor heterogeneity. Recent studies suggest that qualitative methods like MSKCC and modified Healey are effective for assessing MEC, showing superior concordance in grading. In contrast, quantitative systems like AFIP and Brandwein show lower concordance, with Brandwein often assigning higher grades and AFIP lower grades, which could impact treatment decisions [25–27].

In non-neoplastic salivary tissue, OCT4 exhibited positive cytoplasmic reactions in the ductal epithelium of normal salivary glands and negative expression in mucous acinar cells, which agreed with Destro Rodrigues et al. [28]. This finding implies that cells capable of proliferation and differentiation reside within the ducts and may represent a potent salivary stem/ progenitor cell population [29]. MENA exhibited positive cytoplasmic reactions in the ductal epithelium and serous acini of normal salivary glands, while mucous acinar cells showed negative expression. This suggests that MENA is selectively localized in certain cell types of the salivary glands, potentially indicating a functional role in those specific areas. However, a contrasting study by Alerraqi & Badawy [11] found that all tested normal salivary glands were negative for the anti-MENA antibody. Furthermore, previous studies noted weak expression of MENA in normal tissues adjacent to various cancers, suggesting that overexpression entails pathologic alteration [19–21].

In the current study, the positive immunoreaction for OCT4 was detected in the cytoplasm of intermediate and epidermoid cells, and a negative expression in mucocytes. This finding is consistent with Alexander et al. [30] and Jing et al. [31], who reported the cytoplasmic expression of OCT4 without nuclear immunoreaction. Although earlier literature primarily described nuclear immunoexpression of OCT4, more recent studies by Choi et al. [32], Jiang et al. [33], and You et al. [34] found that OCT4 was mainly expressed in the cytoplasm of tumor cells in glioblastoma, gastric, and rectal respectively. The variation of OCT4 subcellular localization among various studies may be attributed to different factors, including binding partners, protein isoforms, and the presence or absence of nuclear localisation or nuclear export signals [35]. The presence of different isoforms of OCT4 protein, specifically OCT4A and OCT4B, which are generated through alternative splicing. These isoforms exhibit distinct subcellular localization patterns: OCT4A is primarily found in the nucleus, while OCT4B is predominantly located in the cytoplasm [35]. OCT4A is responsible for maintaining the stemness characteristics of CSCs. It is crucial for the self-renewal and pluripotency of these cells, which are essential for tumor initiation and progression. Meanwhile, OCT4B promotes tumor growth by inhibiting apoptosis, enhancing angiogenesis, allowing tumor cell migration to the surrounding tissue, and inducing epithelial-to-mesenchymal transition (EMT) [34, 36–38]. Moreover, OCT4 is a polyclonal and non-specific antibody as monoclonal. The study found no significant correlation between OCT4 expression and clinical data or histopathological grades, aligning with previous research [7, 8, 39, 40]. Although no statistically significant correlation was found between positive immunohistochemical expression of OCT4 and the histopathological grades of MEC, immunoreactivity varied among the different grades of MEC. Strong expression predominated in low-grade MEC, whereas negative or weak expression was more common in high-grade MECs. These findings suggest that OCT4 may play a role in carcinogenesis. Similar studies in cervical cancer and OSCC indicated that while increased OCT4 expression is linked to early tumor progression through inhibition of apoptosis, it is not useful for predicting later-stage disease or prognosis [8, 40]. In contrast, Ghazi et al. [41] reported a significant correlation between OCT4 expression and age and histopathological grade in OSCC. Other studies have shown that high OCT4 expression is associated with poor prognosis, including high-grade tumors, advanced stage, metastasis, and lower survival rates [23, 34, 42].

In MEC, MENA shows cytoplasmic and membranous brown staining in epidermoid and intermediate cells in all MEC cases but is absent in mucocytes, consistent with prior studies identifying these locations as key intracellular sites of MENA activity [19, 43]. During EMT, MENA undergoes alternative splicing events, wherein downregulation of the non-metastatic Mena11a isoform occurs concomitantly with upregulation of the invasive MenaINV variant, potentiating metastatic capability [16, 44]. Overexpression of MENA was linked to tumor progression, invasion, and poor prognosis in various cancers [19, 21, 45]. In this study, MENA expression showed significant associations with age, tumor size, and TNM staging, consistent with previous findings in gastric carcinoma, indicating that higher MENA levels may reflect more advanced disease states and larger tumor sizes [20]. Also, there was a significant association between MENA expression and histopathological grades, with higher expression in high-grade MEC, suggesting its role in cellular dedifferentiation, tumor progression, and increased metastatic potential. MENA may serve as a biomarker for predicting the aggressive behavior of MEC. These results were consistent with Hu et al. [45], who reported similar correlations in hepatocellular carcinoma. However, Alerraqi & Badawy [11] found no statistically significant correlation between immunohistochemical expression for MENA and MEC grades, as all of their MEC cases showed strong positive cytoplasmic expression for MENA.

Even more, the Spearman correlation test revealed a weak, non-statistically significant correlation between OCT4 and MENA, as it seems to be a spurious relation. while the Wnt/β-catenin pathway potentially interconnects these proteins, alternative signalling mechanisms, including TGF-β, may modulate their expression independently [5, 12, 17, 18, 46–48]. Cellular and molecular heterogeneity of MEC and variations in the MEC microenvironment might influence the expression of OCT4 and MENA differently.

The need of data regarding recurrence, perineural invasion, necrosis, distant metastasis, and follow-up would enable more prognostication. We could not find rare patterns of MEC (warthin-like MEC, spindled MEC, trabecular MEC, ciliated MEC or pigmented MEC) to correlate the morphology with IHC expression. Also, a complete analysis of the *OCT4* gene isoforms molecularly may have defined specific splicing variants in MEC and their role.

## Conclusion

MENA may serve as a biomarker for predicting MEC’s aggressive behavior. While OCT4 expression may play a role in the early stages of carcinogenesis, it is not useful for predicting later-stage disease or prognosis. The Wnt/β-catenin signaling pathway does not significantly link OCT4 and MENA, suggesting the presence of additional layers of regulation, such as epigenetic modifications, post-translational modifications, or crosstalk with other signaling pathways, which complicate the simple relationship between OCT4 and MENA.

## Electronic Supplementary Material

Below is the link to the electronic supplementary material.

Supplementary Material 1

## Data Availability

Data is provided within the manuscript or supplementary information files.
