# Supplementary figures and images for: OCT4 and MENA immunoprofiling in salivary mucoepidermoid carcinoma
Source: Diagn Pathol. 2025 May 27;20:67. doi: 10.1186/s13000-025-01665-8 (PMC12108025; doi:10.1186/s13000-025-01665-8)

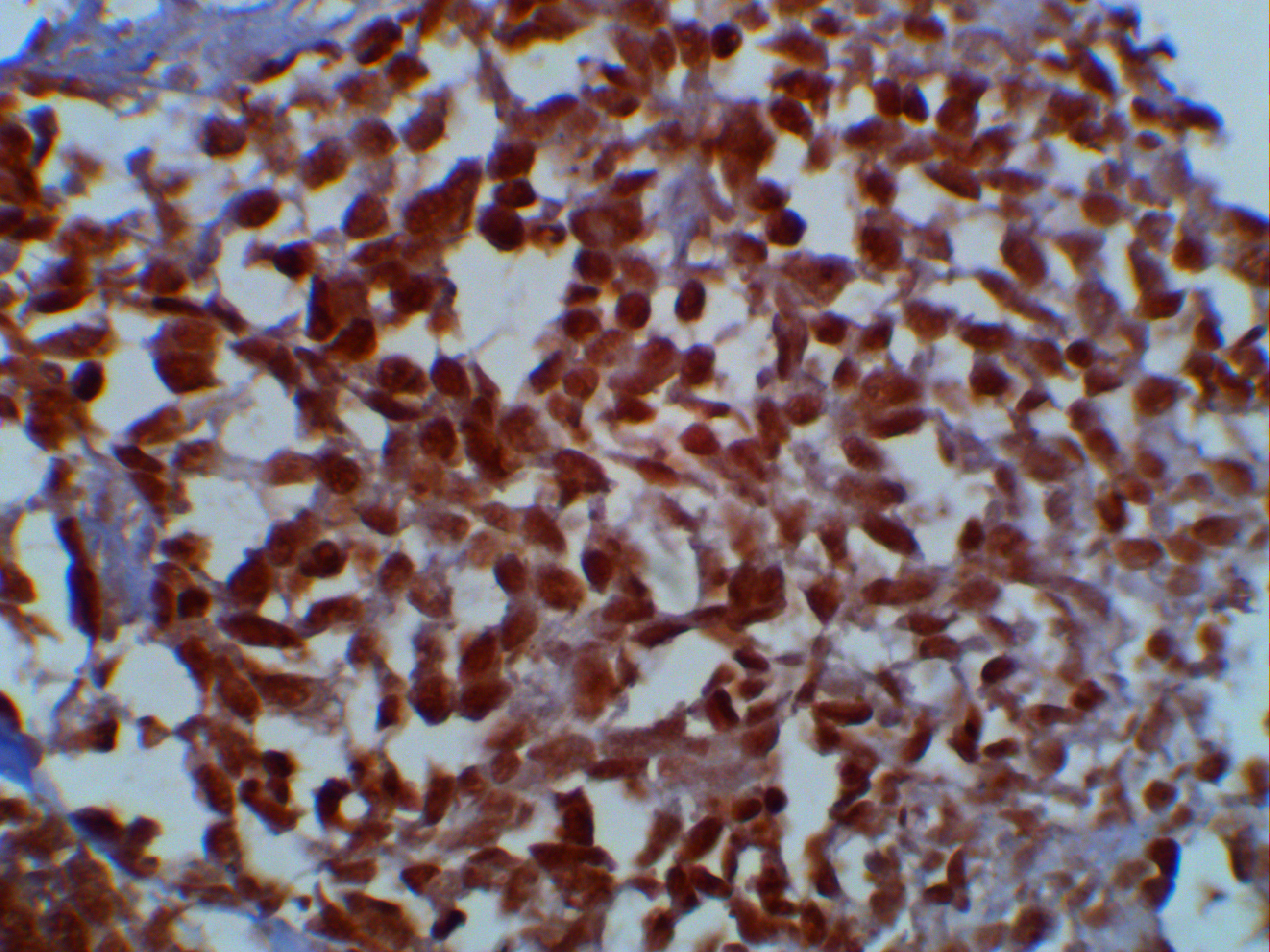

Supplement: Supplementary file 1 — Supplementary Material 1 [file 13000_2025_1665_MOESM1_ESM.zip › Control 2 IHC/control-40x-100 oct4.png]

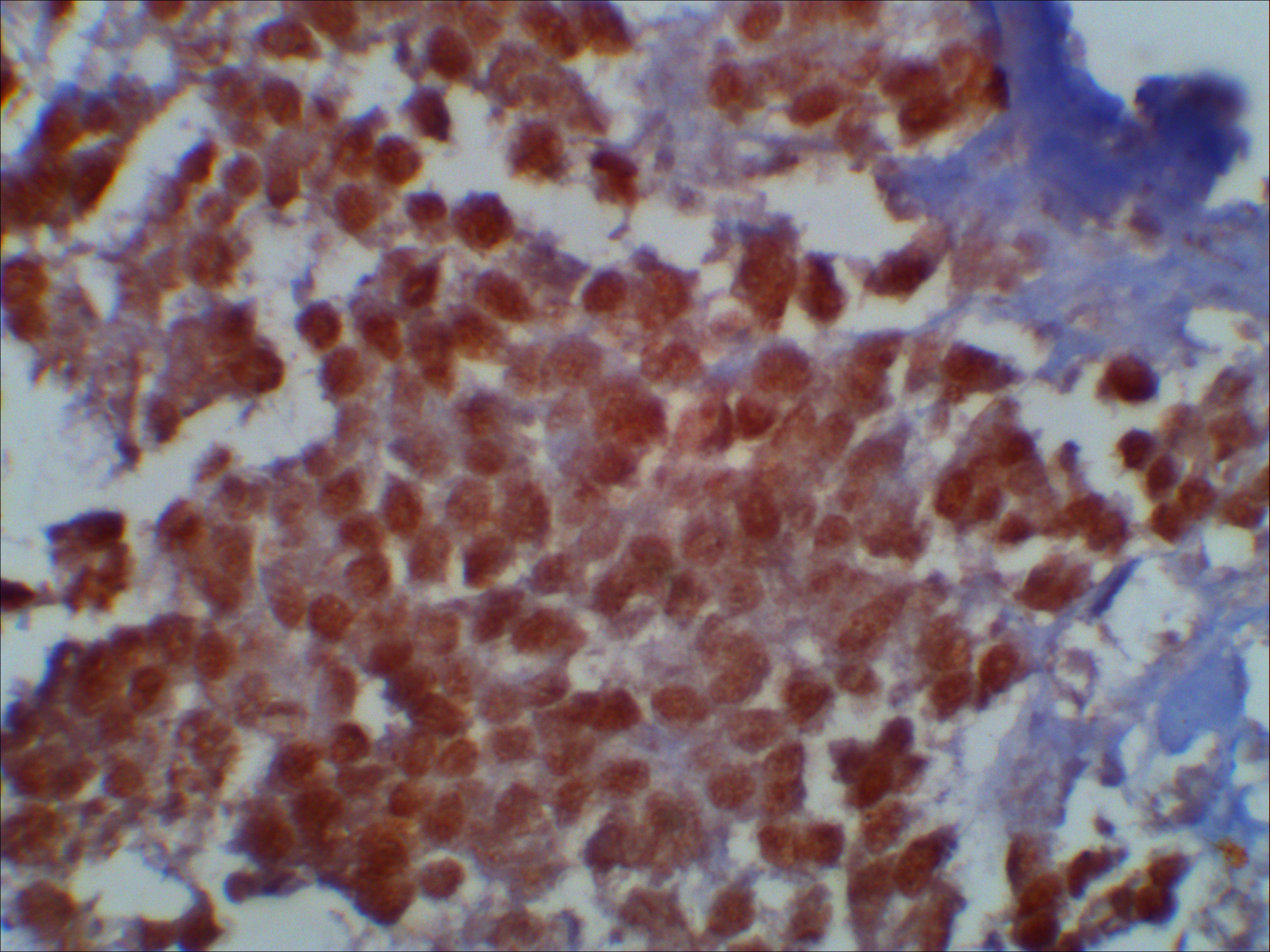

Supplement: Supplementary file 1 — Supplementary Material 1 [file 13000_2025_1665_MOESM1_ESM.zip › Control 2 IHC/control-40x-75 oct4.png]

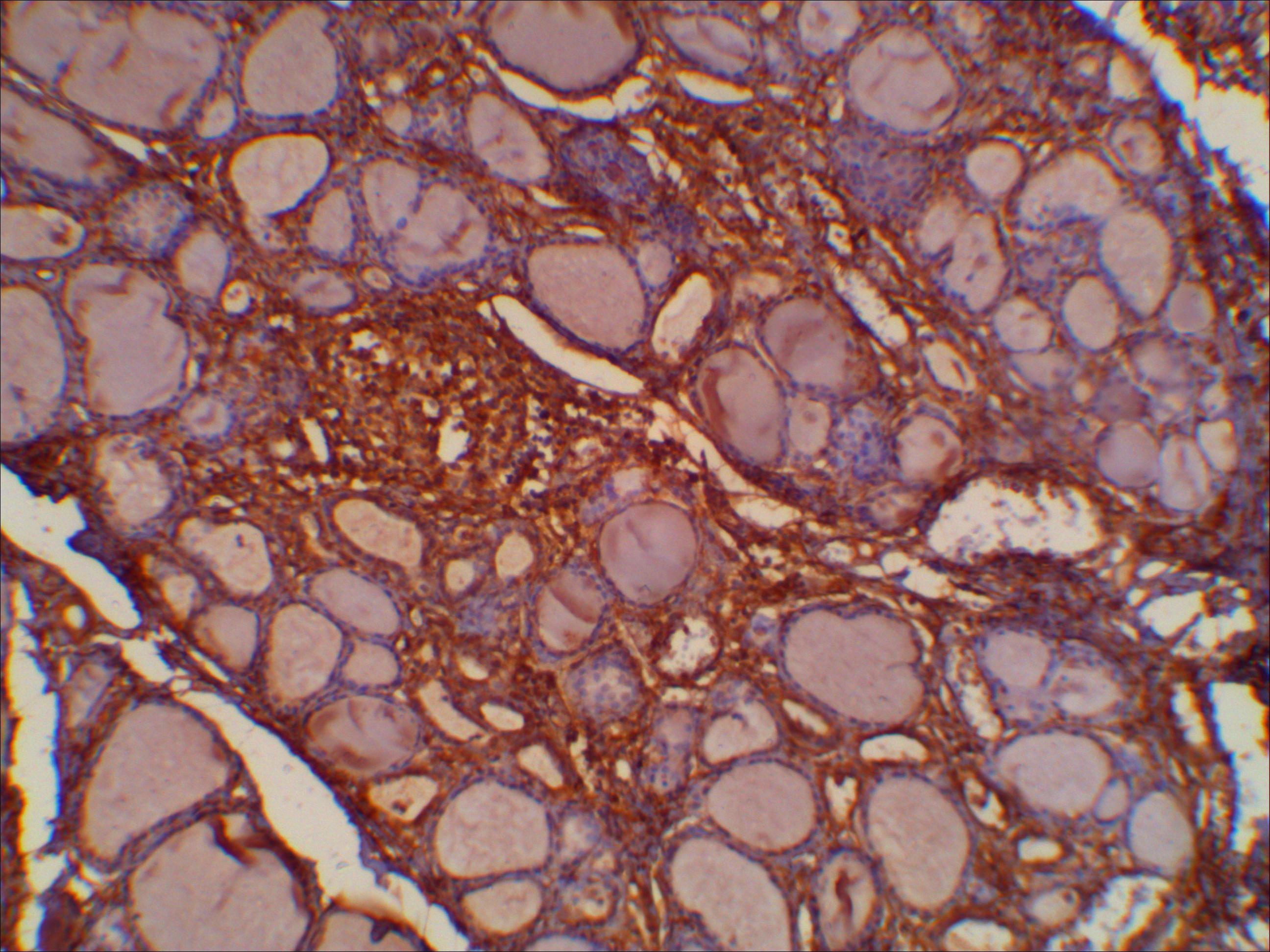

Supplement: Supplementary file 1 — Supplementary Material 1 [file 13000_2025_1665_MOESM1_ESM.zip › Control 2 IHC/ENAH-CONROL-10X.jpg]

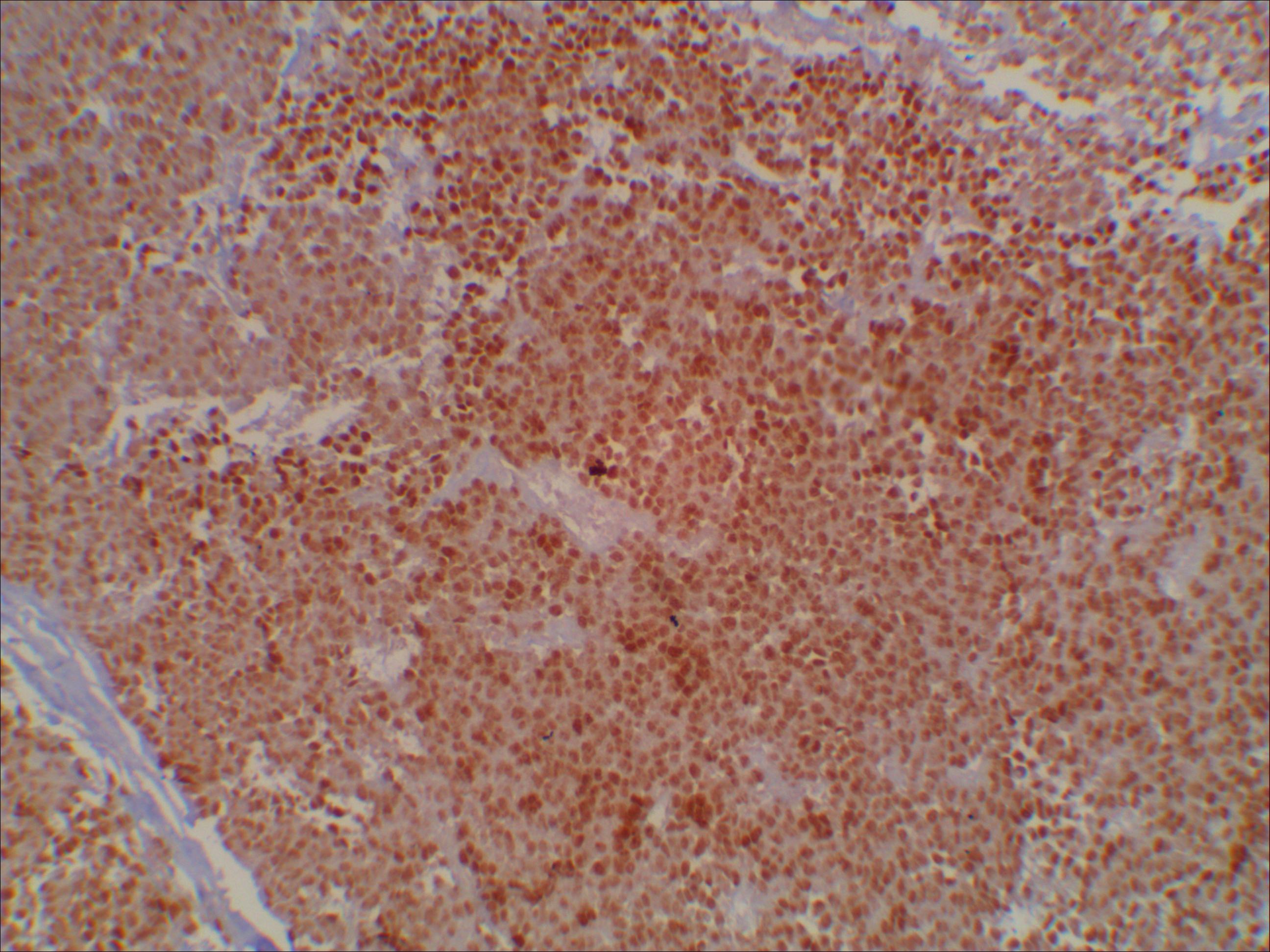

Supplement: Supplementary file 1 — Supplementary Material 1 [file 13000_2025_1665_MOESM1_ESM.zip › Control 2 IHC/OCT4-CONTROL-10X.jpg]
